# Effect of a short-term water-based exercise program on cognitive skills in a Mexican older adult population

**DOI:** 10.3389/fnhum.2025.1593969

**Published:** 2025-08-07

**Authors:** Ermilo Canton-Martinez, Iván Rentería, Juan Pablo Machado Parra, Rubén Avilés-Reyes, Javier Arturo Hall-López, Paulina Yesica Ochoa-Martínez, Patricia Concepción García Suárez, José Moncada-Jiménez, David K. Johnson, Alberto Jiménez Maldonado

**Affiliations:** ^1^Facultad de Deportes, Universidad Autónoma de Baja California, Ensenada, México; ^2^Facultad Ciencias Administrativas y Sociales Universidad Autónoma de Baja California, Ensenada, Mexico; ^3^Department of Exercise and Sport Sciences, New Mexico Highlands University NMHU, Ensenada, Mexico; ^4^Department of Health, Sports and Exercise Sciences, University of Kansas, Lawrence, KS, United States; ^5^Human Movement Sciences Research Center (CIMOHU), University of Costa Rica, San José, Costa Rica; ^6^Department of Neurology, University of California, Davis, Davis, CA, United States

**Keywords:** executive functions, older adults, memory, land-based exercise, water-based exercise

## Abstract

**Introduction:**

Aging leads cognitive decline, affecting memory, attention, and executive functions. Physical exercise, particularly aerobic exercise, enhances cognitive function and health in older adults. Similarly, aquatic-based exercise improves cardiovascular fitness, muscle strength, and cognitive performance in acute- and long-term programs. The present study evaluated the effect of 4-week aquatic based exercise program on the cognitive function of older adults.

**Methods:**

Sixteen older Mexican adults were assigned into an aquatic-based exercise (*n* = 9; 67.9 ± 6.9 years) or land-based exercise (*n* = 7; 66.8 ± 6.9 years) training groups. Verbal and visual memory were assessed via Rey Auditory Verbal Learning Test (RAVLT) and Rey Osterrieth Complex Figure test (ROCF). Executive functions for inhibitory control, cognitive flexibility, and verbal fluency were measured with the five-digit test (FDT) and the Controlled Oral Word Association Test (COWAT) respectively.

**Results:**

Encoding and recall performance improved significantly following both interventions as recalled on RAVLT scores (*p* < 0.05). Favorable changes in visual memory were also observed for both groups with higher delayed recall scores once completed the exercise programs (*p* < 0.05). On the other hand, no significant changes in executive functions by either exercise intervention were observed.

**Discussion:**

Aerobic exercise performed for 4 weeks may improve memory domains, regardless of the type of exercise practiced, while executive functions do not appear to be affected. The data obtained in the current work suggest that short-term interventions which involved aerobic exercise are feasible to improve the memory in older Mexican adults.

## 1 Introduction

Aging is a natural process that inevitably leads to adverse changes in brain morphology and function (Brito et al., 2023; Lee and Kim, 2022). Cognitive deficiencies, such as visual and verbal memory, attention, and executive functions (e.g., information processing, reaction time, inhibitory control, and working memory) are examples of the different domains affected by this process (Brito et al., 2023; Erickson et al., 2010). Erickson et al. (2010), reported an inverse relationship between spatial memory performance and left hippocampal volume (Erickson et al., 2010). Similarly, Persson et al. (2006) observed smaller hippocampal size in older adults with deficits in episodic memory (Persson et al., 2006). Moreover, the authors showed that these impairments are often accompanied by activation of other brain regions in comparison with healthy age-matched controls, possibly as a compensatory mechanism (Persson et al., 2006). Others reported that poor inhibitory control is closely related to dysfunction between the putamen and cortical brain connectivity in older adults, as manifested in the Stroop task (Fjell et al., 2017). Furthermore, slower processing speed and lower executive function scores, including fluency, working memory, and cognitive flexibility are associated with reduced striatal volume (Hedden et al., 2016, 2012).

Cognitive decline in these domains significantly affects the quality of life in older adults (He et al., 2023; Stites et al., 2018). To mitigate this issue, healthcare professionals, researchers, and international organizations strongly advocate for the regular practice of physical activity (Erickson et al., 2019; ACSM, 2022). Research have demonstrated that older adults who engage in moderate-to-vigorous physical activity (MVPA) present superior memory performance with sedentary elderly (Erickson et al., 2019). Additionally, muscle strength is positively linked with a working memory performance (Liao et al., 2024).

In consideration with the above, the benefits of regularly practicing aerobic exercise (e.g., walking, cycling), resistance training, and other activities commonly performed on land are emphasized by the general consensus (Erickson et al., 2019; Liao et al., 2024). However, some older adults face challenges engaging in land-based exercise, mostly due to joint conditions (e.g., arthritis), excess body weight, or the fear of falling (Aquatic Exercise Assosiation, 2018). To address these limitations, alternatives such as water-based exercises (WBE) are commonly suggested (Aquatic Exercise Assosiation, 2018). Water properties, like viscosity, hydrostatic pressure, and temperature provides benefits to minimize fall risk, joint strain, and body weight bearing due to buoyancy in WBE (Simas et al., 2017; da Silva et al., 2019).

Consequently, WBE interventions have been associated with multiple health benefits, including reductions in systolic blood pressure (Neiva et al., 2018), improvements in the lipid profiles and oxidative stress markers (da Silva et al., 2019), benefits on cardiorespiratory fitness (Bocalini et al., 2010), and increased upper and lower limb strength (Neiva et al., 2018; Bocalini et al., 2010; Bento-Torres et al., 2019). Additionally, studies have demonstrated that WBE positively affects brain function in older adults (Bento-Torres et al., 2019; Lima et al., 2015; Wang et al., 2020; Cancela Carral and Ayán Pérez, 2008). For instance, long-term participation in WBE has been linked to improved general cognition performance and a faster reaction times to visual stimuli compared to land-based exercise or sedentary lifestyle (Bento-Torres et al., 2019; Wang et al., 2020; Cancela Carral and Ayán Pérez, 2008; Farinha et al., 2021). Moreover, cognitive functions such as executive function, attention, and memory have shown acute improvements following five consecutive days of aquatic exercise (Fedor et al., 2015). Despite these promising findings, further research is needed to characterize the effects of programs with varying durations and to determine the optimal dose of physical activity for enhancing cognitive function in older adults (Liao et al., 2024; Gallardo-Gómez et al., 2022). Additionally, studies focusing on the efficacy of physical exercise in Latin American older populations remain limited (Jimenez-Maldonado, 2024). Therefore, the present study aimed to evaluate the impact of a short-term water-based exercise program on cognitive function in older Mexican adults.

## 2 Materials and methods

### 2.1 Participants

Sixteen older adults (65.0 ± 8.8 years) were recruited for this study. All participants were regular attendees of the “Active Aging” community health program at the Facultad de Deportes of Universidad Autónoma de Baja California. All participants were provided with detailed information about the study's objectives and procedures before the enrollment. Each participant read and signed an institutionally-approved consent form. The following inclusion criteria were chosen: 1) An official proof of complete COVID-19 vaccination, including booster doses, 2) Medical clearance from their healthcare provider or from a recognized public health institution, confirming that they are free from musculoskeletal or cardiovascular conditions that could prevent them from safely engaging in physical activity sessions, whether conducted on land or water, 3) Familiarization with swimming was not considered an inclusion criterion. Whereas, the exclusion criteria were the follow: 1) Have suffered of a cardiovascular event or neurological disease with or without mental or cognitive disorders, 2) Participants who reported chronic alcohol consumption, and 3) Have any skin infection, the latter is considered a potential risk of transmission to other participants during aquatic interventions. The medical report provided essential information about the health status of the participant (e.g., diagnosed with diabetes, hypertension, metabolic syndrome, etc.), from this official report we classified the individuals into comorbid or healthy categories for demographic purposes. All participants successfully completed the intervention.

### 2.2 Study design

The quasi-experimental study employed a pre-test/post-test design. After the study's objectives and procedures were explained, participants freely selected their preferred exercise modality. As a result, nine individuals enrolled in the water-based exercise group (8 females, 1 male; 67.9 ± 6.9 years), while the land-based exercise group consisted of seven women (66.8 ± 6.9 years).

This study was conducted in accordance with the principles of the Helsinki Declaration and was reviewed and approved by the institutional review board of *Comité de Ética y Evaluación de Investigación y Posgrado (CEEIP)* of the *Facultad de Deportes, Ensenada* campus, at *Universidad Autónoma de Baja California*. The study was registered under the code CEEIP0001-FDE.

### 2.3 Exercise programs

The WBE program incorporated aerobic and resistance exercises, conducted in a structured pool environment. Sessions were held three times per week over 4 weeks, lasting 60 min each session. The pool depth ranged from shoulder to waist level for most participants, and the water temperature was maintained at an average of 30°C to ensure comfort for older adults. The volume and frequency of the land-based exercise program was similar to WBE. The specific exercises performed in water- and land-based sessions are detailed in Table 1.

**Table 1:** Exercise session in the water and land-based exercise.

**Exercise phases**	**Duration**	**Water-based exercise (WBE)**	**Land-based exercise**
Warm-Up Phase	10 min	Controlled arm circles (10 repetitions per side)	Controlled arm circles (10 repetitions per side)
		Ankle rotations (10 repetitions per side)	Ankle rotations (10 repetitions per side)
		Gentle neck movements (5 repetitions per direction)	Gentle neck movements (5 repetitions per direction)
		Recovery Interval: 15–20 s between exercises	Recovery Interval: 15–20 s between exercises
Training	40 min		
Aerobic conditioning	20 min	Water walking or jogging: 3 min per set/3 sets/30-s recovery	Water or jogging: 3 minutes per set/3 sets/30-s recovery
		High knee lifts (skipping motion): 15 repetitions per leg/3 sets/20-s recovery	High knee lifts (skipping motion): 15 repetitions per leg/3 sets/20-s recovery
		Heel lifts (butt kicks): 15 repetitions per leg/3 sets/20-s recovery	Heel lifts (butt kicks): 15 repetitions per leg/3 sets/20-s recovery
		Aqua-aerobics or Aqua-Zumba routines: 10 min of continuous activity	Zumba-Dance routines: 10 min of continuous activity
		Water walking or jogging: 3 min per set/3 sets/30-s recovery	Water walking or jogging: 3 min per set/3 sets/30-s recovery
		High knee lifts (skipping motion): 15 repetitions per leg/3 sets/20-s recovery	High knee lifts (skipping motion): 15 repetitions per leg/3 sets/20-s recovery
		Squats performed with water resistance: 12 repetitions/3 sets/30-s recovery	Squats performed with chair: 12 repetitions/3 sets/30-s recovery
Muscular resistance training	20 min	Arm movements mimicking swimming strokes: 15 repetitions/3 sets/20-s recovery	Pec Deck/Pullover/Cable Fly: 15 repetitions/3 sets/20-s recovery
		Hip extension, abduction, and adduction movements: 12 repetitions per leg/3 sets/30-s recovery	Hip extension, abduction, and adduction movements: 12 repetitions per leg/3 sets/30-s recovery
		Lateral steps and frontal pushes: 10 repetitions per side/3 sets/20-s recovery	Lateral steps and frontal pushes: 10 repetitions per side/3 sets/20-s recovery
		Heel raises: 15 repetitions/3 sets/20-second recovery	Heel raises: 15 repetitions/3 sets/20-s recovery
		Squats performed with water resistance: 12 repetitions/3 sets/30-s recovery	Squats performed with chair: 12 repetitions/3 sets/30-s recovery
Cool-down and recovery phase	10 min	Slow, controlled movements to promote relaxation of major joints: 5 repetitions per exercise	Slow, controlled movements to promote relaxation of major joints: 5 repetitions per exercise
		Recovery Interval: 15–20 s between exercises	Recovery Interval: 15–20 s between exercises
		Gentle stretching targeting the primary muscle groups engaged during the session: 20 s	Gentle stretching targeting the primary muscle groups engaged during the session: 20 s

### 2.4 Cognitive skills measurements

The general cognitive ability (GCA) was measured using the Montreal Cognitive Assessment (MoCA), a validated tool for Mexican populations (Aguilar-Navarro et al., 2018). The learning and verbal memory were assessed with the Rey Auditory Verbal Learning Test (RAVLT; Bell et al., 2020). In the RAVLT, the examiner readed out loud a list of 15 nouns (List A) and asked participants to recall as many words as possible (free recall). This procedure was repeated for five consecutive trials (trials 1–5). Following this, a second list of 15 nouns (interference or List B) was presented, and participants were asked to recall as many words as possible from List B. Immediately after, delayed recall of List A was tested without presenting the list again (trial 6). After a 30-min delay, participants were asked again to recall words from List A (delayed recall, trial 7). Lastly, a recognition task was administered where participants listened to a list of 50 nouns, including words from Lists A and B and 20 phonologically or semantically similar words. The participants were tasked to identify all the words that had appeared in List A (Magalhães and Hamdan, 2010; Miguel Sánchez-Nieto et al., 2016). The number of correct words recalled in each trial for List A was recorded to assess learning and verbal memory. Additionally, the correct number of recalled words from List B, immediate recall, (delayed words from List A), and long-term verbal memory (delayed recall from List A) were also recorded (Bell et al., 2020). To reduce the familiarization effects, the RVALT included an alternate forms during the post intervention assessment (Calamia et al., 2012).

Inhibitory control and cognitive flexibility (executive functions members), were measured using the five-digit test (FDT), following procedures outlined in previous studies (Paiva et al., 2016; de Paula et al., 2011; Sedó García-Tuñón, 2004). The FDT consists of two sections. The first section demands simple cognitive processing tasks (reading and counting), and the second involves more complex tasks (inhibition and shifting). In the reading section, the examiner presents 50 cards with boxes containing congruent digits (e.g., one 1, two 2, three 3, etc.), which participants must read aloud. In the counting section, participants count the number of asterisks (ranging from 1 to 5) on each card (e.g., ^**^ “two”).

The inhibition section, which incorporates the Stroop effect, presents incongruent scenarios: cards with groups of digits that does not correspond with their arithmetic values, in this section the participant must count the number of digits in each group (e.g., “3-3-3-3”, with the correct answer being “four” instead of “three”). The shifting section adds cognitive flexibility by introducing a new rule. Stimuli are highlighted to prompt the participant to shift from counting to reading the numerals (e.g., “4-4-4” should now be answered as “four” instead of “three”). To ensure comprehension, participants underwent a brief training session consisting of 10 stimuli for each section of the FDT. The FDT scores are based on the time (in seconds) taken to complete each section, with shorter times indicating better cognitive performance (Sedó García-Tuñón, 2004). The number of errors made during each task serves as an index of accuracy (Calamia et al., 2012).

The verbal fluency (VF) was assessed using the Controlled Oral Word Association Test (COWAT), referred to in Spanish as “Fluencia de letra inicial” (Buriel et al., 2004). During the COWAT, participants were instructed to generate as many words as possible within 1 min that began with the letter “A.” This task was followed by two semantic fluency tests, in which participants were asked to names as many animals and fruits as possible within the same time constraint (Chávez-Oliveros et al., 2013). Additionally, a word exclusion task was administered, requiring participants to generate words that did not contain the letter “E” (VFWE) or “S” (VFWS), with each trial lasting 1 min. The selected letters were chosen based on their frequency in Spanish language (Buriel et al., 2004). The VF domain score was determined by counting the number of correct words produced in accordance with task rules, as well as the number of errors committed (Buriel et al., 2004; Chávez-Oliveros et al., 2013).

The Rey Osterrieth Complex Figure (ROCF) test was used to assess immediate and delayed visual memory recall, following a previously established methodology (Lunge et al., 2000; Meyers and Meyers, 1995). In this test, participants were presented with a sheet of paper containing a printed copy of the ROCF in the top half of the page and were instructed to replicate the figure in the space below as accurately and quickly as possible (ROCFT-1). Upon completion, the copied figure was removed, and participants were asked to reproduce the figure from memory on a blank sheet of paper (immediate recall, ROCFT-2). After a 30-min interval, they were again asked to recall and draw the figure from memory (delayed recall, ROCFT-3).

### 2.5 Statistical analysis

The statistical analysis was performed with the IMB SPSS statistic version 21.0 (IMB-SPSS statistic, Armonk, NY, USA). The descriptive data statistics are reported as the mean and standard deviation (M ± SD). Additionally, the median, central tendency, and first and third quartiles were calculated as measures of dispersion for cognitive variables. To assess between groups differences before and after the intervention, the Mann-Whitney test was applied. Within-group differences across time points were analyzed using the Wilcoxon test. Statistical significance was set at *p* < 0.05.

## 3 Results

Demographic data are presented in Table 2, while the cognitive domains outcomes are reported in Table 3. Regarding general cognition, MoCA unexpectedly suggested that participants in the aquatic exercise group may be experiencing mild cognitive impairment (MCI). However, baseline GCA values did not differ between groups (Table 3).

**Table 2:** Major sample characteristics at baseline groups *n* = 16.

**Characteristics**	**Land-based exercise**	**Water-based exercise (WBE)**	***P=***
Sex (M/W)	0/9	1/6	
AGE (years)	67.11 ± 4.19	66.71 ± 5.70	0.8747
BMI (kg/m^2^)	28.41 ± 5.07	28.01 ± 3.96	0.8674
Years of education	11.11 ± 3.89	11.21 ± 5.89	0.938
**RMED**			
Yes (%)	*N =* 6/66.67%	*N =* 6/85.71%	
No (%)	*N =* 3/33.33%	*N =* 1/14.29%	
Diabetes (%)	*N =* 1/11.11%	*N =* 1/14.29%	
Hypertense (%)	*N =* 2/22.22%	*N =* 2/28.57%	
Comorbid (%)	*N =* 3/33.33%	*N =* 3/42.86%	
Healthy (%)	*N =* 3/33.33%	*N =* 1/14.29%	
Total sample per group	*N =* 9/100%	(7) 100%	

Body Mass Index (BMI); Regular Medication (RMED), clinically diagnosed as diabetic and hypertensive patients (Comorbid), clinically identified as healthy, Male/Women (M/W). Results are presented in number of participants, Mean, Standard Deviation and percentage.

**Table 3:** Neurocognitive variables for water and land-based exercise.

**Variables**	**Water-based exercise (WBE)**						**Wilcoxon**	**Land-based exercise**						**Wilcoxon**	**Mann-Whitney U**			
	**PRE**			**POST**				**PRE**			**POST**				**PRE (Water vs. Land)**		**POST (Water vs. Land)**	
	**Q1**	**Median**	**Q3**	**Q1**	**Median**	**Q3**	***p***	**Q1**	**Median**	**Q3**	**Q1**	**Median**	**Q3**	***p***	***U***	***p***	***U***	***p***
MOCA	18.20	**22.00**	26.25	17.75	**24.00**	24.75	0.036^*^	22.00	**25.00**	27.00	22.00	**26.00**	28.00	0.032^*^	60.00	0.129	83.50	0.713
RAVLT 1	2.00	**3.50**	4.00	3.75	**5.00**	6.00	0.026^*^	2.50	**4.00**	5.00	3.00	**5.00**	5.50	0.052	76.00	0.456	88.50	0.901
RAVLT 2	4.75	**6.50**	7.00	4.75	**7.50**	9.25	0.114	5.00	**6.00**	7.00	6.00	**7.00**	7.50	0.040^*^	79.50	0.569	83.50	0.713
RAVLT 3	4.75	**7.50**	9.25	6.00	**10.00**	11.25	0.033^*^	6.50	**8.00**	9.00	7.00	**8.00**	12.00	0.323	77.50	0.508	80.00	0.589
RAVLT 4	6.50	**8.00**	11.00	7.50	**9.00**	14.00	0.0490^*^	8.00	**8.00**	11.00	9.00	**10.00**	11.50	0.033^*^	89.50	0.942	80.00	0.589
RAVLT 5	6.50	**9.50**	11.50	7.75	**11.50**	15.25	0.002^*^	8.00	**10.00**	13.00	9.00	**11.00**	13.00	0.349	77.50	0.509	70.00	0.305
List B	2.75	**4.00**	5.00	3.00	**4.00**	4.00	0.524	3.00	**4.00**	4.50	2.50	**4.00**	5.00	0.943	82.50	0.672	90.50	0.980
FR List B	4.50	**7.00**	9.00	5.00	**10.00**	10.00	0.042^*^	5.00	**8.00**	10.00	6.00	**9.00**	11.50	0.228	75.50	0.449	66.50	0.231
FR Delayed RAVLT	4.50	**6.50**	9.00	3.00	**7.00**	11.00	0.474	5.00	**7.00**	9.00	6.50	**10.00**	14	0.036^*^	84.00	0.732	64.50	0.196
FDT reading (errors)	0.00	**0.00**	0.25	0.00	**0.00**	0.00	0.109	0.00	**0.00**	0.00	0.00	**0.00**	0.00	1.00	71.50	0.083	91.00	1.00
FDT reading (time)	25.75	**31.50**	40.50	25.75	**33.50**	37.50	0.937	26.00	**36.00**	45.50	25.50	**28.00**	35.50	0.278	80.50	0.610	75.50	0.450
FDT counting (errors)	0.00	**0.00**	0.50	0.00	**0.00**	0.00	0.109	0.00	**0.00**	0.00	0.00	**0.00**	0.00	0.317	71.50	0.083	84.00	0.299
FDT counting (time)	30.00	**32.00**	38.75	27.00	**30.00**	35.00	0.151	26.50	**32.00**	38.50	28.50	**34.00**	39.00	0.889	81.50	0.644	69.00	0.284
FDT choosing (errors)	0.00	**1.50**	2.00	0.00	**0.00**	1.25	0.041^*^	0.00	**1.00**	2.50	0.00	**0.00**	1.50	0.234	84.50	0.743	86.00	0.784
FDT choosing (time)	39.75	**45.00**	54.00	38.75	**44.00**	50.25	0.141	40.00	**45.00**	52.50	40.00	**45.00**	51.00	0.574	89.50	0.942	84.00	0.734
FDT shifting (errors)	0.00	**1.00**	3.00	0.00	**0.50**	1.00	0.285	0.50	**2.00**	5.00	0.00	**1.00**	5.00	0.531	64.00	0.180	68.50	0.246
FDT shifting (time)	48.75	**53.50**	67.25	46.75	**56.00**	67.00	0.826	50.50	**54.00**	75.00	49.50	**62.00**	74.00	0.834	73.50	0.395	72.00	0.356
COWAT “A”	10.00	**11.50**	15.50	7.50	**11.50**	18.50	0.729	8.00	**14.00**	16.50	9.50	**13.00**	17.50	0.964	90.00	0.961	85.50	0.789
COWAT “A” (errors)	0.00	**0.50**	1.25	0.00	**0.00**	1.25	0.876	0.00	**1.00**	1.50	0.00	**1.00**	2.00	0.667	73.50	0.365	69.00	0.257
COWAT animals (score)	13.75	**15.50**	17.75	13.75	**17.00**	20.75	0.267	14.00	**17.00**	19.00	13.50	**19.00**	21.50	0.622	79.50	0.571	90.50	0.981
COWAT animals (errors)	0.00	**1.00**	1.25	0.00	**1.00**	2.00	0.507	0.50	**2.00**	3.50	0.00	**0.00**	1.00	0.095	61.50	0.139	80.50	0.583
COWAT fruit (score)	15.75	**17.50**	20.00	17.75	**18.50**	20.00	0.128	14.50	**19.00**	20.00	17.00	**18.00**	22.50	0.014^*^	85.00	0.769	78.00	0.522
COWAT fruit (errors)	0.00	**2.00**	4.00	0.75	**1.50**	3.25	0.822	1.00	**1.00**	3.50	1.00	**2.00**	3.00	0.722	86.50	0.824	79.00	0.553
VFWE (score)	7.00	**11.50**	14.00	8.75	**10.00**	12.50	0.799	8.50	**10.00**	13.50	8.00	**9.00**	13.00	0.906	90.50	0.981	88.00	0.883
VFWE (errors)	0.00	**1.00**	2.25	0.00	**1.00**	5.25	0.346	1.00	**2.00**	4.00	0.0000	**0.00**	3.00	0.154	59.50	0.117	67.00	0.215
VFWS (score)	8.75	**12.00**	14.25	7.75	**14.00**	15.25	0.598	9.00	**12.00**	16.50	10.50	**12.00**	14.50	0.969	81.50	0.641	90.50	0.981
VFWS (errors)	0.00	**1.00**	2.00	0.00	**1.50**	4.00	0.299	0.00	**1.00**	3.00	0.00	**1.00**	2.50	0.905	85.00	0.763	74.00	0.397
ROCFT 1	22.00	**27.00**	31.50	31.50	**35.00**	37.00	0.015^*^	26.50	**32.00**	33.50	30.50	**35.00**	35.50	0.028^*^	84.00	0.733	89.50	0.941
ROCFT 1 time (sec)	188.75	**232.00**	310.25	187.00	**250.00**	321.00	0.331	138.50	**210.00**	247.00	140.00	**161.00**	225.00	0.272	68.00	0.264	38.00	0.010^*^
ROCFT 2	6.50	**16.00**	21.25	12.00	**19.50**	27.50	0.023^*^	16.50	**21.00**	28.25	15.50	**22.00**	28.00	0.701	51.50	0.054	77.50	0.512
ROCFT 2 time (sec)	114.00	**174.00**	203.25	127.50	**170.50**	251.25	0.272	128.00	**170.00**	196.50	123.00	**158.00**	201.00	0.507	85.50	0.789	75.50	0.452
ROCFT 3	8,775	**11.50**	19.25	10.75	**22.00**	24.50	0.031^*^	15.00	**17.00**	19.50	16.50	**21.00**	28.50	0.016^*^	77.00	0.495	70.50	0.038^*^
ROCFT 3 time (sec)	110.75	**137.50**	183.50	82.25	**116.00**	162.50	0.166	112.50	**149.00**	190.00	99.00	**120.00**	160.00	0.099	85.50	0.790	87.50	0.865

Montreal Cognitive Assessment MOCA (Score), Beck Depression Inventory (BDI). Depression (score), Rey Auditory Verbal Learning Test (RAVLT), Free-Recall (FR). Five-Digit Test (FDT), Controlled Oral Word Association Test (COWAT), Verbal Fluency Without “E” word (VFWE), Verbal Fluency Without “S” word (VFWS), Rey Osterrieth Complex Figure (ROCF).

In terms of cognitive improvements, verbal memory improved in both groups. However, executive functions including inhibitory control and cognitive flexibility, remained unchanged following either exercise program, and VF did not exhibit significant changes post intervention (Table 3).

For visuospatial function and visual memory, the ROCF test revealed that the WBE improved the direct copying score, and the performance linked with the immediate and delayed recall. In contrast, the land-based exercise group showed improvements in direct copying and delayed recall, but not in the immediate recall (Table 3).

## 4 Discussion

The present study demonstrated that both exercise modalities led to improvements in general cognition, verbal memory, and visual memory (delayed recall), cognitive domains primarily regulated by the hippocampus (Ekstrom, 2014). In contrast, executive function domains remained unaffected by either exercise program.

Although none of the participants had clinical diagnosis of mild cognitive impairment, MoCA scores suggested that individuals in the WBE group might meet the criteria for mild cognitive impairment (MCI; cutoff score <25; Aguilar-Navarro et al., 2018; INGER, 2022). In contrast, those in the land-based exercise group exhibited MoCA scores indicative of normal cognitive function. Despite these cutoff values, baseline statistical analyses revealed no significant differences in GCA between the two groups. Previous studies have reported that a 12-month aerobic exercise intervention can improve GCA in healthy older adults (Muscari et al., 2010). However, a recent meta-analysis found that moderate-intensity exercise did not significantly affect GCA (Taylor et al., 2019; Northey et al., 2018; Leckie et al., 2014). Separate works from Northey et al. (2018), and Sanders et al. (2019) consistently found a high heterogeneity observed in these studies may account for the lack of a significant effect (Taylor et al., 2019; Sanders et al., 2019). Additionally, these published meta-analyses have primarily compared exercise groups to control groups (between-group analysis) rather than assessing cognitive changes within the same participants over time (within-group analysis), which could contribute to the discrepancies between our findings and those of previous research (Northey et al., 2018; Sanders et al., 2019).

On the other hand, verbal and visual memory significantly improved following the completion after 4 weeks of moderate-intensity exercise programs (Table 3), aligning with previous findings from WEB (Wang et al., 2020; Farinha et al., 2021; Fedor et al., 2015) and land-based exercise interventions (Taylor et al., 2019). Visual memory is highly dependent on hippocampal function (Erickson et al., 2010, 2011), and the cognitive benefits observed may be attributed to exercise-induced neurobiological adaptations in this brain region.

Moderate-intensity exercise has been shown to upregulate biomolecules associated with brain plasticity, such as brain-derived neurotrophic factor (BDNF), insulin-like growth factor-1 (IGF-1; Wang et al., 2020; Kim and Kim, 2018), and irisin (Kim and Kim, 2018; Küster et al., 2017). Increased energy expenditure promotes neurotrophin synthesis in the hippocampus (Wrann et al., 2013; Novelle et al., 2013). Since the increased physical activity in our experimental groups contributed to the cumulative energy expenditure throughout the intervention, we can infer that repeated exercise sessions in our study participants elevated hippocampal molecules, like irisin, subsequently enhancing BDNF expression in the hippocampus and improving memory performance.

Furthermore, Arazi et al. (2021) reported a combined effect of strength and endurance exercise on these memory-related molecules (i.e., BDNF and IGF-1). Regarding IGF-1 and exercise, discussing its impact on memory in the long term can be controversial, with opinions leaning from trivial toward significant effects (Stein et al., 2018; He et al., 2023). Our exercise protocols involved strength training, a modality known to increase circulating IGF-1 levels in older adults (Arazi et al., 2021). Although we did not measure growth factor concentrations during the study, we cannot rule out the possibility that their increased levels may infer some accountability in memory function following the interventions (Sonntag et al., 2013; Torres-Aleman, 2010).

In addition to the potential molecular adaptations discussed above, previous studies have reported that a single session of moderate-intensity water-based exercise can enhance cerebral blood flow (CBF; Parfitt et al., 2017; Pugh et al., 2015; Carter et al., 2014), a physiological response associated with improved cognitive performance. Therefore, it is plausible that repeated exercise sessions in water contribute to sustained increases in CBF, ultimately enhancing memory task performance in our participants. Moreover, non-strenuous or non-exhaustive exercise has been shown to increase the arousal system, which is linked to improved verbal memory performance (Schmidt-Kassow et al., 2014). This suggests an additional mechanism by which exercise may facilitate verbal memory improvements in our participants.

In contrast, the cognitive domains related to executive function remained unchanged regardless of the exercise modality. Specifically, semantic and phonological VF did not show statistically significant improvements following the intervention; our data are consistent with findings of previous meta-analyses (Turner et al., 2021; Bir et al., 2021). VF is a complex executive function associated with frontal lobe activity that requires mental flexibility, rapid information retrieval, response initiation, and inhibitory control (Tyburski et al., 2015; Henry and Crawford, 2004). While VF is influenced by multiple factors, it is primarily associated with frontal lobe function. inhibitory control (Tyburski et al., 2015; Henry and Crawford, 2004).

Prior research has highlighted the role of education level to modulate VF performance (Chávez-Oliveros et al., 2013). Our participants reported an average of 11 years of education, with some having attained less than high school level. This relatively low educational background may have contributed to the lack of improvement in VF after the intervention. A possible neurobiological explanation for this association is the concept of cognitive reserve, where lower educational attainment is linked to reduced cognitive resilience and adaptability (Suemoto et al., 2022; Lövdén et al., 2020).

The present study's findings indicate that the short-term exercise programs did not significantly impact inhibitory control or cognitive flexibility. This result contrasts with previous studies, which employed longer interventions (21 weeks) compared with the current study (−4 weeks; Albinet et al., 2016, 2010), these longer interventions (~21 weeks) reported improvements in executive function, highlighting the importance of exercise duration in eliciting cognitive changes (high order cognitive domains).

Prior research has suggested that improvements in executive function may be influenced by vagal activity (Albinet et al., 2016, 2010). However, evidence indicates that the parasympathetic system is less responsive to exercise than the overall autonomic nervous system in older adults (Raffin et al., 2019). This phenomenon led us to suggest that short-term water—or land-based aerobic exercise programs may be insufficient to induce meaningful changes in vagal activity and, consequently improve the executive function performance.

This study has several limitations. First, the absence of a control group necessitates caution with the interpretation of the results. Therefore, studies employing randomized controlled trials (RCTs) are needed to validate our initial findings. Second, molecular and physiological markers related to cognitive function were not assessed, preventing definite conclusions about the mechanisms underlying the observed improvements in memory. Lastly, the small sample size increases the risk of type II errors, potentially limiting the generalizability of the findings.

## 5 Conclusion

These findings suggest that short-term (4 week) aerobic exercise interventions are effective in enhancing memory. This cognitive domain is particularly significant, as impaired memory performance is an independent risk factor for the development of Alzheimer's disease (Caselli et al., 2014). Furthermore, the results underscore the potential benefit of short-term, moderate-intensity exercise programs in preserving cognitive function among older Latino adults.

## Data Availability

The raw data supporting the conclusions of this article will be made available by the authors, without undue reservation.
